# In Situ Swelling Formulation of Glycerol-Monooleate-Derived Lyotropic Liquid Crystals Proposed for Local Vaginal Application

**DOI:** 10.3390/molecules27196295

**Published:** 2022-09-23

**Authors:** Martine Tarsitano, Antonia Mancuso, Maria Chiara Cristiano, Donatella Paolino, Massimo Fresta

**Affiliations:** 1 Department of Health Science, University “Magna Græcia” of Catanzaro, 88100 Catanzaro, Italy; 2 Department of Experimental and Clinical Medicine, University “Magna Græcia” of Catanzaro, 88100 Catanzaro, Italy

**Keywords:** vaginal mucosa, in situ forming formulation, sertaconazole nitrate, cubic phase precursor, spontaneous degradation, Kinexus^®^ Pro rheometer

## Abstract

Hydrogels have been extensively investigated to identify innovative formulations that can fulfill all the necessary purposes to improve local vaginal therapy through the mucosa. Herein, we propose in situ-forming lyotropic liquid crystals (LLCs) derived from a cheap and GRAS (generally recognized as safe) ingredient as an intravaginal delivery system. The system consists of a precursor solution loaded with sertaconazole nitrate as a model drug, which is able to easily swell in a stable three-dimensional structure by absorbing simulated vaginal fluid. Under polarized light microscopy the precursor solution and the formed phase of LLCs showed the typical textures belonging to anisotropic and an isotropic mesophases, respectively. A deep rheological investigation by Kinexus^®^ Pro proved the stability and strength of the cubic phase, as well as its potential in mucoadhesion. In vitro degradation studies showed a slow matrix erosion, consistent with data obtained from lipophilic drug release studies in simulated vaginal fluid. Therefore, the suggested cubic phase based on lyotropic liquid crystals could represent a valid proposal as a vaginal drug delivery system due to its characteristics of resistance, adhesion and the possibility of providing a slow and controlled release of drugs directly at the administration site.

## 1. Introduction

The oral administration of active molecules, for example, for the treatment of vaginal candidiasis, is easy and comfortable but not without negative effects and consequences that can even become serious. Hepatic first-pass effects, high incidence and severe gastrointestinal side effects, and the request of high drug doses can occur following oral drug administration [1].

As a result of these claims, attempts have been increasingly made to use more suitable administration routes for the treatment of local diseases. This is the case for the treatment of sexually transmitted vaginal diseases and other female local infection. For such pathologies, the vaginal administration route could be the most suitable; however, the physioanatomic structure of the vagina makes it not particularly easy to achieve therapeutic efficacy. Although the vagina is characterized by an extended permeation area and rich vascularization, the absorption of drugs after intravaginal administration can be highly variable as a function of formulation properties, physicochemical features of the drug, local physiology, age of the patient, menstrual period and the possible presence of wounds [2,3].

Considering that it is not possible, in many cases, to address most of these factors, the choice of formulation plays a key role in achieving therapeutic success with maximum patient comfort. The ideal vaginal formulation should be easy to self-administer and discreet, such as a solution or a suspension; of reversible application, such as a stimuli-responsive gel; able to adhere to mucosa; and cost-effective, painless and safe [4]. Moreover, a minimum persistence time on the vaginal mucosa is required to ensure drug release and the efficacy of treatment [5].

In our opinion, the best formulation characterized by all of these features could be represented by a precursor solution, fluid and sliding, that becomes a three-dimensional matrix, solid-like, layered and sufficiently adhesive only after administration and only after interacting with the wet vaginal environment. These features are provided by so-called lyotropic liquid crystals, namely systems with a structural organization that is influenced by the amount of solvent and not by other stimuli; on the contrary, when the maximum hydration state is achieved, the resulting three-dimensional network is highly stable as a function of pH, temperature or light variations [6,7].

Lyotropic liquid crystals are characterized by amphiphilic domains that can contain within their structures both hydrophilic and hydrophobic drugs, establishing more or less strong bonds. The bonds between drug(s) and lyotropic liquid crystals affect the release profile and, in some cases, the degradation profile of the system. The main structures of lyotropic liquid crystals are lamellar and cubic phases, and the transition from one phase to another is induced exclusively by the presence of excess moisturizing medium. A lamellar phase can be considered the precursor solution of the cubic phase, as it is more fluid and is characterized by a linear order of lipid bilayers. In this arrangement, the alkyl chains are organized toward the center of the layers, whereas the polar portion of the constituent amphiphilic lipid faces the external aqueous compartment, enabling these three-dimensional sheets to slide over each other. When the lamellar phase is exposed to excess hydrating medium, a more stimuli-resistant, solid-like structure, called the cubic phase, is formed. This last phase is constituted by tortuous networks of aqueous nano-channels responsible for sustained release of drugs from liquid crystals [8].

Herein, we propose lyotropic liquid crystals (LLC), the swelling of which is triggered by aqueous medium absorption, as a resistant, easy-to-administer and cheap drug delivery system for vaginal application. To characterize the proposed systems, we used sertaconazole as a model drug, which is already used for vaginal disease treatments [9,10,11] and present in various conventional dosage forms, such as tablets, solutions and creams [12]. Compared to conventional dosage forms, the proposed lyotropic liquid crystals should be able to persist longer on the vaginal mucosa, ensuring a slow and controlled release of the active ingredient. To this end, we investigated the ability of the precursor solution to transform itself into a cubic phase, as well as the rheological and optical behavior of the achieved LLCs. Furthermore, we evaluated the stability, mucoadhesion potential, degradation and release profiles of formulations by mimicking the vaginal environment.

## 2. Results and Discussion

### 2.1. Transition from Precursor Solution to Cubic Phase

**Figure 1:**
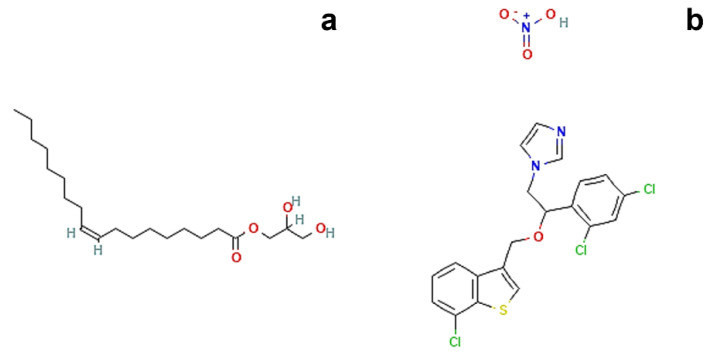
Chemical structures of (**a**) Glycerol monooleate. Available online: https://pubchem.ncbi.nlm.nih.gov/compound/5283468#section=2D-Structure (accessed on 19 September 2022, [15]) and (**b**) Sertaconazole nitrate. Available online: https://pubchem.ncbi.nlm.nih.gov/compound/200103#section=2D-Structure (accessed on 19 September 2022, [16]).

The main component of the proposed formulations is glycerol monooleate (GMO, Figure 1a), an amphiphilic polar lipid well known in the scientific community as a GRAS (generally recognized as safe) material and widely used in various drug delivery contexts [6,13].

It belongs to the class of water insoluble lipids, which are able to swell in aqueous media and to form distinct LLCs. This process is driven by the self-assembly of GMO molecules that orient the polar heads and the hydrophobic hydrocarbon tails in different three-dimensional structures according to the amount of water present [14].

Among these structures, the lamellar phase and the cubic phase represent the two opposite poles, as these two conformations are characterized by completely different spatial distributions and rheological behavior. The percentage of water present in the system is the determining factor for the transition from the lamellar phase to the cubic phase, attributing to each phase the ability to exhibit liquid or crystalline solid properties. These distinctive physicochemical properties make LLCs suitable for solubilizing and delivering both oil- and water-soluble compounds [17].

Therefore, in the present study, a precursor solution of the cubic phase was prepared by choosing 80:20 as the fixed ratio (% *w*/*w*) between GMO and water; then, a transition to the cubic phase was performed by adding an excess of aqueous medium. The same operative conditions were employed during the preparation of precursor solution loaded with sertaconazole nitrate (Figure 1b), a highly lipophilic drug (logP = 6.23) characterized by extremely poor aqueous solubility [18,19].

**Figure 2:**
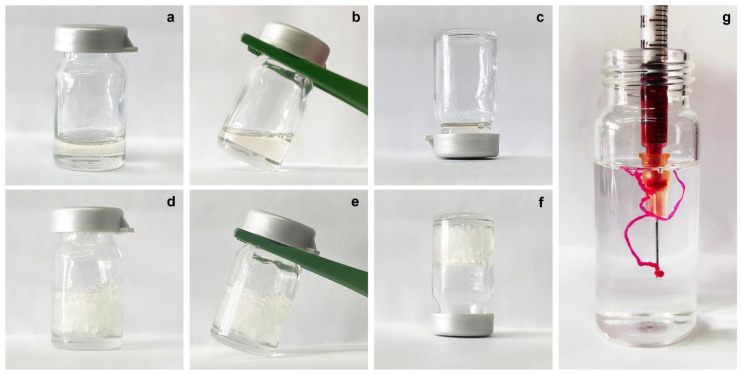
Representative photographs of the transition process from (**a**–**c**) precursor solution to (**d**–**f**) fully swollen cubic phase according to the test-tube inversion method; (**g**) instantaneous transition of precursor solution when injected in SVF with a 25G insulin syringe. The reported photographs refer to the drug-loaded formulations, as no macroscopic differences occurred with respect to the empty formulations.

For these reasons, the drug was solubilized in GMO in advance to start the drop-by-drop hydration. Once both precursor solutions (with and without drug) were obtained, the samples were fully characterized, starting with the phase transition test. The incorporation of the model drug did not alter the formulation, as both precursor solutions appeared transparent and slightly viscous but free to flow, as shown in Figure 2a–c. To mimic the physiological milieu of the human vagina, a simulated vaginal fluid (SVF, pH 4.2) was employed for a phase transition test of the precursor solutions, as well as for further experiments [20,21].

First, the transition to the cubic phase was conducted by adding an excess of SVF directly to the glass tube containing the precursor solution to ensure a complete swelling of the system, which was confirmed by a simple inverse flipping of the vial. Hence, the formed cubic phases appeared as homogenous and highly viscous, milky samples, which were no longer able to flow (Figure 2d–f).

To prove the instantaneous transition of precursor solution into cubic phase when surrounded by an aqueous environment, the sample, dyed with Nile red to visualize the transition, was directly injected into an SVF-filled vial by means of an insulin syringe. No difficulties arose in withdrawing the precursor solution, despite the use of a 25G needle, and as soon as the free-flow sample came into contact with the SVF, it lost its flowability, transitioning into a steady cubic phase (Figure 2g). This leads us to suppose the ease of in situ administration of the formulation, even through the use of devices with larger diameter dispensers, such as those commonly used for topical vaginal administration. Furthermore, the precursor solution would spontaneously self-assemble into the LLC cubic phase when surrounded by physiological vaginal fluid, without the need for other external chemical or physical stimuli to trigger its transition in situ, like other previously proposed ionically or covalently crosslinked hydrogels [22,23].

The addition of sertaconazole nitrate to the formulation did not alter the ability of the precursor solution to swell and form an LLC cubic phase, maintaining the same macroscopic aspect as the empty formulation. This must be taken into consideration, as the addition of any component to the formulation (e.g., a drug) could affect the transition process and the aspect of the obtained cubic phase, as well as its properties [24].

**Figure 3:**
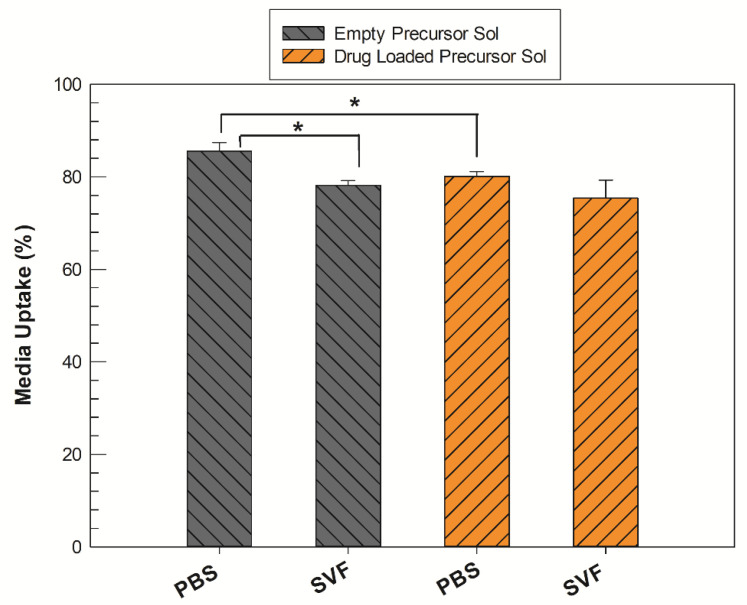
Medium uptake (%) of precursor solutions (Sol) in their transition to fully swollen cubic phases, both in PBS and SVF solution. The gray bars represent the empty samples, whereas the orange bars refer to drug-loaded samples. The results are expressed as mean values of three independent experiments ± standard deviation, * *p* < 0.05.

The exact amount of aqueous medium that the precursor solution was able to absorb in its three-dimensional structure during swelling was recorded by gravimetric method using both PBS (pH 7.4) and SVF (pH 4.2) solutions to determine any differences in swelling ability of precursor solutions with and without the addition of the drug. The uptake percentage values (%) of each medium are reported in Figure 3.

All samples were able to absorb more than 75% of aqueous medium with respect to their initial weight, regardless of environmental pH values. In PBS, the precursor solution containing sertaconazole required a significantly lower amount (* *p* < 0.05) of aqueous medium to form a stable LLC cubic phase compared to the empty analogue. When the test was replicated in SVF medium, the empty precursor solution also inflated itself with a lower medium uptake percentage with respect to that obtained in PBS, whereas there was no significant difference between medium uptake from drug-loaded precursor solution samples, even if the pH and composition of the media differed.

The clearest emerging data trend is that regardless of the medium used, when the drug is present in the system, the formation of the cubic phase occurs by absorbing a reduced quantity of water. This event could be ascribable to the presence of sertaconazole nitrate. By its nature, the drug could localize itself inside the alkyl chain domain of a GMO-derived LLC structure, increasing the hydrophobicity of the system and thus reducing the precursor solution’s ability to accommodate water molecules and make them reside within its network [25].

### 2.2. Morphological Investigation of LLCs

As the implementation of formulations with additional components can modify the physical characteristics of the resulting cubic phase, this event also has a non-negligible impact on the LLCs’ three-dimensional structures. There is a strong interest among researchers in the effects deriving from the incorporation of additives in LLC formulations, as this could involve changes in the rheological behavior of mesophases, favor the transition from one phase to another or even enable them to coexist [26,27].

To distinguish them from external factors that can alter the phases (e.g., temperature, pressure, light and magnetic field), the presence of amphiphilic molecules, the water content and the addition of third additives are indicated as prescription factors [28].

**Figure 4:**
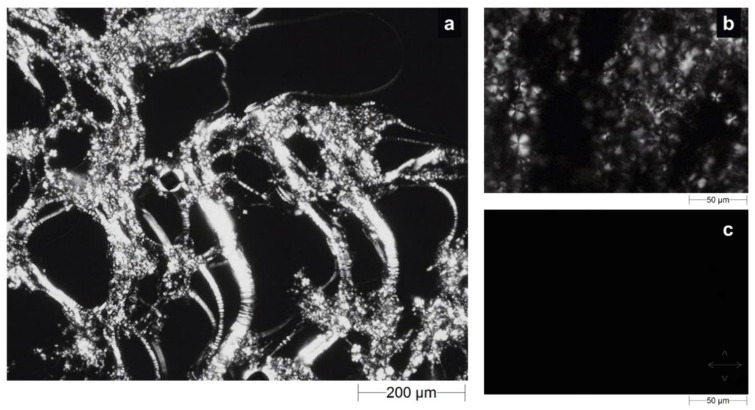
Optical micrographs obtained by CPLM analysis of the drug-loaded precursor solution at (**a**) 5× and (**b**) 20× magnifications and the corresponding cubic phase (**c**) obtained after swelling in SVF. Crossed white double arrows indicate the orientation of polarizer filters.

We morphologically investigated the precursor solutions and water-swollen LLC cubic phase by crossed-polarized light microscopy (CPLM) analysis to classify samples based on the corresponding observed texture. This method represents a gold-standard analysis to identify lamellar, hexagonal or cubic phases, as each phase may or may not show a typical texture when exposed to polarized light, depending on its birefringence characteristics. Under polarized light, lamellar and hexagonal phases are anisotropic and show Maltese crosses, also known as cruciate flowers, and “fan-like” textures, respectively. The cubic phase is isotropic, so it does not possess optical birefringence properties [29,30,31]. When the drug-loaded precursor solution was observed under polarized light, a “streaky” texture with cruciate flowers appeared on a black background, as shown in Figure 4a,b.

On the contrary, when the fully swollen LLC cubic phase was examined, the birefringent texture disappeared, giving way to a completely black background (Figure 4c) as a result of the final cubic phase. Effectively, the presence of cruciate flowers combined with the streaky texture is an unmistakable confirmation of the predominance of a lamellar phase structure in the precursor solution. However, this characteristic texture with optical properties of birefringence was alternated with dark areas typical of an isotropic material, such as the cubic phase, suggesting the coexistence of the phases. This seems to agree with the previously reported medium uptake results and is supported by the theory that the incorporation of lipophilic drugs, such as sertaconazole nitrate, drives the transition from lamellar to cubic phase [28,32].

The absence of a “fan-like” texture led us to exclude the possibility that a hexagonal phase cohabited with the lamellar phase in precursor solution, whereas the absolute absence of light signals during the analysis of the swollen formulation confirmed the formation of a pure cubic phase [33].

### 2.3. Stability Analysis and Rheological Behavior Studies

#### 2.3.1. Precursor Solutions Stability over Time

**Figure 5:**
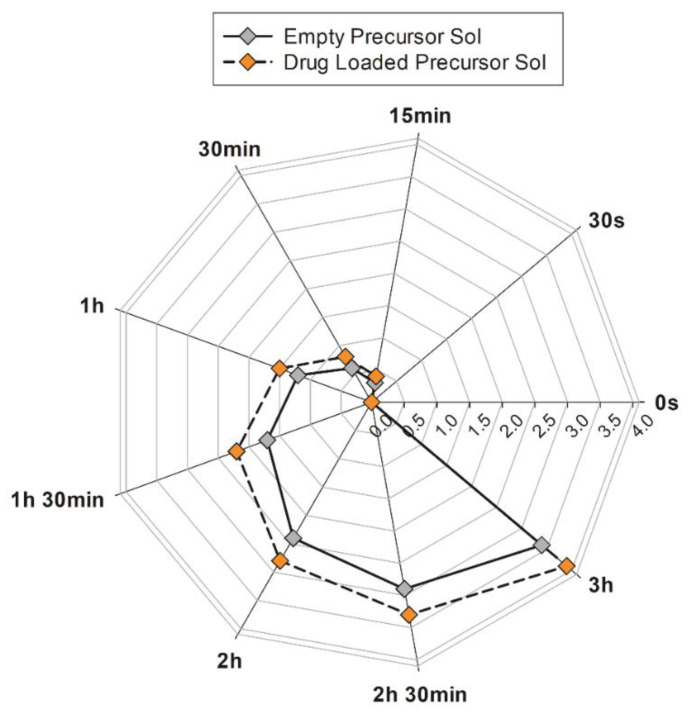
TSI values recorded by Turbiscan^®^ Lab Expert during long-term stability study of precursor solutions with (orange symbols) or without (grey symbols) drug addition. The results are representative of five independent experiments. The mean values ± standard deviation are available in Table S1 of Supplementary Materials.

Although LLCs are characterized by a high solubilization capacity for hydrophilic, lipophilic and amphiphilic guest drug molecules [6,34], and during the preparation of samples, no macroscopical sediments or phase separation were observed, a long-term stability test was performed on precursor solutions, either loaded or not with sertaconazole nitrate. The analysis consisted of multiple continuous scans by a laser beam that crossed the formulation from the bottom to the top of a glass vial, which recorded any variations in transmitted and backscattered light. For each scan performed, the integrated software set up a Turbiscan stability index (TSI) value, a unique number that reflects the destabilization of a given sample, considering all events that can reduce the stability (e.g., sediment thickness, particle flocculation, phase separation, etc.) [35,36]. TSI values of empty and drug-loaded precursor solutions are reported the radar diagram shown in Figure 5.

The precursor solution containing the drug revealed TSI values that could be superimposed on the empty system in the initial seconds of analysis. Then, these values slightly to deviated from the empty precursor solution plot, with the maximum difference in the values never exceeding 0.5. The lower the TSI value, the more stable the formulations are [37].

In summary, in the analyzed samples, the TSI values always remained lower than 4 for as much as 3 h of accelerated analysis. Based on these results, we can conclude that precursor solutions are free from destabilization phenomena in the long term.

#### 2.3.2. Viscosity Measurement with Kinexus Pro^®^

**Figure 6:**
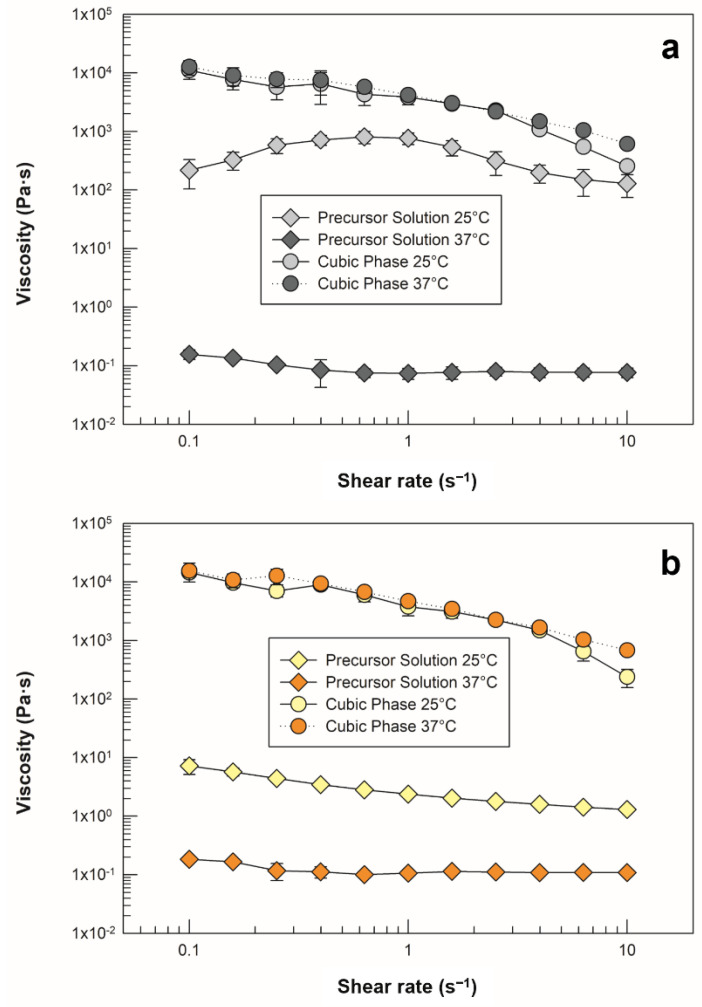
Viscosity profiles of (**a**) empty precursor solution and cubic phase, as well as (**b**) drug-loaded precursor solution and cubic phase. Analyses were carried out at 25 ± 0.5 °C (light grey and light yellow symbols) and 37 ± 0.5 °C (dark grey and orange symbols). The data are reported as mean values ± standard deviation; the error bars, if not shown, are smaller than the symbol size.

Rheological behavior is another feature to consider when discriminating between LLCs. Based on analysis carried out with a rotational rheometer in shear rate mode, the viscosity profiles of empty and drug-loaded precursor solutions and the SVF swollen cubic phase were examined at 25 °C (room temperature) and 37 °C (body temperature). The results are reported in Figure 6.

As previously stated, temperature is a determining external factor with respect to the formation of lyotropic mesophases that is capable of altering their characteristics, as well as the transition among phases. Figure 6a shows that the empty precursor recorded at room temperature did not maintain the same shear viscosity values, and the same trend occurred when the sample was heated and analyzed at body temperature. Therefore, we appraised a predictable strong dependence of this sample on temperature variation, which increased the precursor solution’s ability to flow as a function of heating. As soon as the sample was hydrated with SVF and reached its swelling equilibrium, it lost its fluidity and became highly viscous, as demonstrated by enhanced viscosity values with respect to the precursor solution [38,39].

The maintenance of overlapping shear viscosity plots against the temperature increase (even at 38 ± 0.5 °C, Figure S1) confirmed the formation of a stable, three-dimensional and isotropic cubic phase, which can preserve its structure if administered inside the vaginal canal, where the temperature can be higher than the basal body temperature in the case of pathological conditions, such as bacterial and fungal infections, or as a result of physiological menstrual cycle effect [40].

The same investigation was carried out on the systems in presence of sertaconazole nitrate, and the shear viscosity of the precursor phase was found to be lower at room temperature than that of the empty sample. In particular, a significant reduction in the values was recorded at shear rates below 0.6 s^−1^ (*p* *˂* 0.05) and at a maximum shear rate of 10 s^−1^ (*p* *˂* 0.001).

The drug-loaded precursor solution exhibited shear-thinning behavior, which is in agreement with the results discussed in Section 2.1, as satisfactory syringeability was achieved. No differences were observed in cubic phase analysis, as the plots were superimposable to those of empty samples at both temperatures. Additionally, the resistance of the viscosity to heating was confirmed, supporting the fact that although the presence of the drug modified some characteristics of the precursor solution, its incorporation neither compromised its conversion to cubic phase nor modified its physical stability in response to temperature.

#### 2.3.3. Mucoadhesion Potential of Cubic Phase

Among the parameters that most influence the topical permanence of three-dimensional systems, such as formed in situ hydrogels, the interaction of the formulation with the vaginal cervical mucus is among the most limiting. Mucoadhesive properties support the contact of a drug delivery system with the mucosa covering the vaginal cavity, contributing to the increased residence time at the site of action [41].

The presence of mucin in the intrinsic composition of mucus (as well as water, urea, carbohydrates, salts, proteolytic enzymes and numerous fatty acids) allows for the formation of bonds of various kinds between it and the administered gels, including electrostatic interactions, hydrogen bonds, hydrophobic interactions, etc. [42].

Although GMO itself possesses mucoadhesive properties, as do its derived LLCs, several factors influence this feature, such as the type and the amount of incorporated drug [43,44]. For in vitro investigation of the mucoadhesive properties of the cubic phase, the drug-loaded cubic phase was compared to a mix of the same sample with porcine stomach mucin (PSM) in terms of elastic modulus (G’) values. Oscillatory frequency sweep measurements were conducted after a complete swelling of samples in SVF. The raw data obtained at 0.1, 1 and 10 Hz frequency are reported in Table 1.

**Figure 7:**
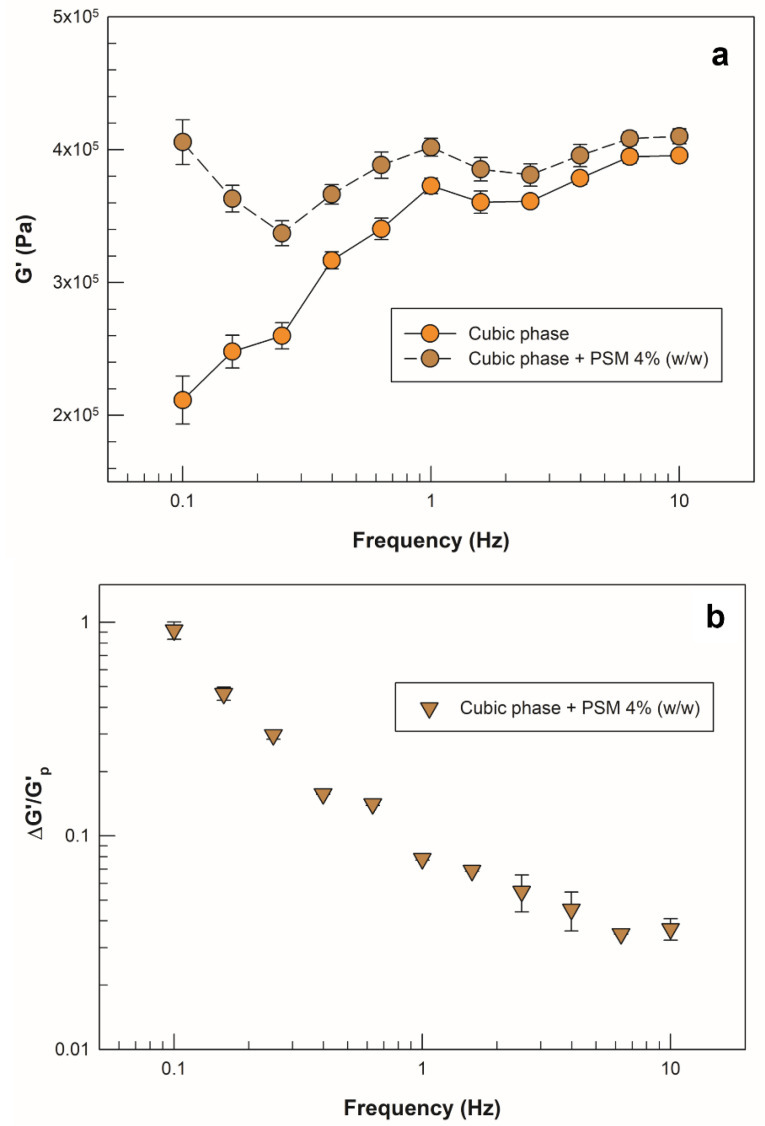
Mucoadhesion assessment with oscillatory frequency sweep analysis. (**a**) Frequency dependence of the elastic modulus (G’) for the drug-loaded cubic phase alone (orange round symbols) and for the mixture with PSM 4% (brown round symbols). (**b**) Frequency dependence of the calculated interaction term (ΔG’/ G’) for the mixture of drug-loaded cubic phase with PSM (brown triangular symbols). All samples were swelled in SVF. The results are reported as mean values ± standard deviation; the error bars, if not shown, are smaller than the symbol size.

The addition of PSM to the cubic phase resulted in positive interaction terms, calculated according to Equation (2) (see Section 3.5), indicating a positive interaction between the cubic phase and mucin. As shown in Figure 7a, the values of the elastic modulus of the mixture with mucin were always greater than in the cubic phase loaded with sertaconazole nitrate alone, and the difference was more marked at lower frequency values. We supposed that the partial positive charge of the drug in solution due to the presence of nitrogen, may contribute to negatively charged mucin upon interaction with electrostatic bonds.

Moreover, because positive interaction terms were recorded, we wanted to investigate the frequency dependence of the relative interaction term (ΔG’/G’_cp_) to understand how the frequency of oscillation could impact the interaction strength. As previously reported, an increase in “solid-like” behavior of a sample in the presence of mucin can be translated into a strong interaction with mucus, and this event could be confirmed by a decrease in relative interaction terms with increasing frequency values [45]. In Figure 7b, the slope of the curve represents this phenomenon, i.e., a linear reduction in the ratio ΔG’/G’_cp_ with increased frequency. This result further supports the potential interaction of the formulation with the mucin present in the vaginal cervical mucus.

### 2.4. In Vitro Degradation and Drug Release Studies in SVF

**Figure 8:**
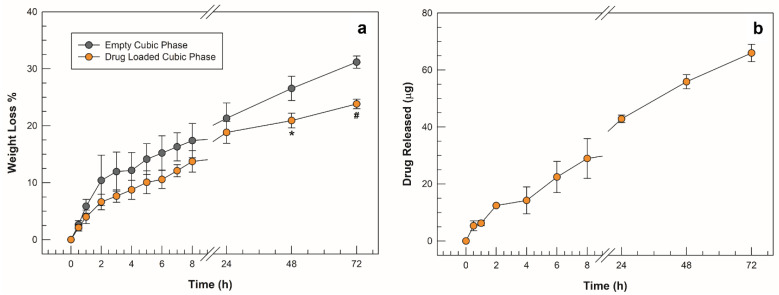
Degradation studies in SVF (**a**) obtained from empty cubic phase (grey symbols) and drug-loaded cubic phase (orange symbols); (**b**) amount of drug released from cubic phase vs. time. The results are expressed as mean values of three independent experiments ± standard deviation, * *p* < 0.05, ^#^ *p* < 0.001.

The cubic phases formed by triggering the swelling with the incorporation of simulated vaginal fluid in their network were subjected to a spontaneous degradation study in the same SVF at pH 4.2 and thermostated at a body temperature of 37 °C. On the basis of the percentage weight loss values obtained from the gravimetric analysis, both the empty cubic phase and the drug-loaded phase showed a high resistance to degradation. Up to 8 h from the setup of the simulation, both the empty formulation and the loaded cubic phase lost less than 20% and slightly more than 10% by weight of their mass, respectively. Overall, the cubic phase loaded with sertaconazole nitrate showed an even slower degradation trend than the empty analogue. The percentage difference became significant after 48 h, as shown in Figure 8a, as the lost weight of the drug-loaded cubic phase was 21 ± 1.3%, whereas the empty cubic phase had already lost 26 ± 2.1% (* *p* < 0.05.). At the end of weight recording after the 72nd hour, the values were around 30% and 23% for the empty and drug-loaded cubic phases, respectively (* *p* < 0.05).

The slower rate of degradation obtained in the presence of the highly lipophilic drug was possibly a result of its deep incorporation into the cubic phase network, which could reduce the ease of access of SVF solution into inner water channels. Theoretically, the slower the degradation of a matrix, the slower the release of the incorporated drug [25], in agreement with the curve of the quantity of drug released from the cubic phase in SVF. A slow and controlled release was recorded, as shown in Figure 8b, and the trend of the release curve mirrors that of degradation. This suggests that, as in previous studies, the kinetics of release of the lipophilic drug from the cubic phase did not occur by diffusion of the drug first in the aqueous channels and then in the release medium but was probably related to the slow but constant erosion of the matrix [34]. To investigate the kinetic mechanism involved in drug release from the cubic phase matrix in depth, the data collected during the in vitro release studies were plotted according to four release kinetic models (zero order, first order, Higuchi and Korsmeyer–Peppas) [46]. Table 2 shows the values of slope and intercept obtained from the regression lines of each model, together with the representative equations, plots and relative correlation coefficient (R^2^).

The Higuchi square root model (R^2^ = 0.9859) was determined to be the best match for drug release, implying that drug release from the matrix is a square-root-of-time-dependent process with diffusion control. However, the actual physiological environment in vivo would change the kinetics of degradation, as well as that of release.

## 3. Materials and Methods

### 3.1. Materials

Glycerol monooleate (GMO, 98 wt% monoglycerides, 1.5 wt% diglycerides and 0.4 wt% free fatty acids) was provided by BASF Catalysts Germany GmbH (Nienburg, Germany). Sertaconazole nitrate, sodium chloride, potassium hydroxide, calcium hydroxide, bovine serum albumin (BSA), lactic acid, acetic acid, glycerol, urea, glucose, porcine stomach mucin (PSM), ethanol and acetonitrile were purchased from Merck (Milan, Italy). Spectra/Por^®^ 50 KDa dialysis membranes were obtained from Spectrum Laboratories (Laguna Hills, CA, USA). Additionally, 1 mL insulin syringes with 25G needles were purchased from Terumo Europe N.V. (Belgium). All other materials and solvents used in this investigation were of HPLC grade. Deionized double-distilled water from a Milli-Q System (Millipore, Milan, Italy) was used throughout the investigation.

### 3.2. Preparation and Phase Transition Test of Precursor Solutions

The precursor solutions were prepared by dropping the appropriate amount of water in preheated GMO (45 ± 0.5 °C) under continuous and vigorous stirring on a heating magnetic stirrer. The drug (0.5% *w*/*w*) was added to the preheated GMO and stirred until it was completely solubilized; then, the hydration started. Both precursor solutions were prepared by maintaining a fixed ratio between GMO and water (8:2 *w*/*w*). The obtained samples were sealed and left to equilibrate overnight away from sources of humidity and heat before continuing with further studies.

The phase transition test was performed by using a simulated vaginal fluid (SVF, pH 4.2) to mimic the vaginal milieu [21]. A volume of 1 mL precursor solution was placed in a glass vial prior to adding an excess of hydrating SVF medium until a complete transition to cubic phase occurred. The SVF was composed of sodium chloride 3.51 (g/L), potassium hydroxide 1.40 (g/L), calcium hydroxide 0.222 (g/L), bovine serum albumin 0.018 (g/L), lactic acid 2.00 (g/L), acetic acid 1.00 (g/L), glycerol 0.16 (g/L), urea 0.40 (g/L) and glucose 5.00 (g/L), and its pH was adjusted to 4.2 with HCL 1 M. The phase transition was checked by the test-tube inversion method [47] and by injecting the precursor solution into 5 mL of SVF with an insulin syringe. The test was performed in triplicate.

### 3.3. In Vitro Simulated Media Uptake Studies

The appropriate amount of SVF absorbed by the precursor solution during its transition to a completely swollen cubic phase was determined by gravimetric method, as reported in our previous study [34], and compared to PBS. Briefly, 1 mL of precursor solution was placed in vial and weighed (M_1_). Subsequently, 5 mL of aqueous medium (PBS pH 7.4 and SVF pH 4.2) was added to the precursor solution. The system was thermostated at 37 ± 0.5 °C to reach the maximum medium uptake and swelling equilibrium. After 30 min, the unincorporated medium was removed, and the beaker containing the swollen cubic phase was weighed (M_2_). The medium uptake percentage (MU%) was calculated according to Equation (1):

MU(%) = [(M_2_ − M_1_)/M_2_] × 100 (1)

### 3.4. Phase Transition Studies

A morphological analysis of both precursor solutions and obtained cubic phases was performed using a Morphologi G3-S microscope (Malvern Panalytical, Malvern, UK) equipped with a Nikon^®^ CFI 60 brightfield/darkfield optical system. A volume of 10 µL of precursor solution (or 10 mg of cubic phase) was gently placed on the instrument plate and immediately covered with a micro cover glass (20 × 20 mm, Syntesys, Italy) to avoid changes in the samples’ degree of hydration. The light source was polarized with cross-polarizing filters (ScreenTech, Germany) and oriented to form a 90° angle. Micrographs were obtained from 5× and 20× magnifications and exported as uncompressed TIFF files by the integrated Morphologi software (Malvern Panalytical, UK).

### 3.5. Rheological Behavior and Mucoadhesion Assessment

The rheological behavior of samples was determined with a Kinexus^®^ Pro rotational rheometer (Malvern Panalytical, UK). The measurements were carried out at 25 ± 0.5 °C and 37 ± 0.5 °C using the cone–plate geometry (diameter 40 mm; angle 2°) for precursor solutions and the plate–plate geometry (diameter 20 mm; gap between geometries of 1.2 mm) for cubic phases. A steady shear-rate sweep analysis was performed to record the viscosity of both the precursor solutions and swollen cubic phases as a function of shear rate from 0.01 to 10 s^−1^.

To evaluate the interaction between cubic phase and mucin, frequency sweep measurements were conducted on a porcine stomach mucin (PSM) solution (4% *w*/*w*), ordinary cubic phases and cubic phases mixed with 4% (*w*/*w*) PSM after their full swelling in SVF. For the analysis, a setup from 0.1 to 10 Hz of frequency and a controlled shear stress of 1.0 Pa were fixed within the linear viscoelastic region (LVER) of each sample to record the values of elastic modulus (G’). Because the G’ of the mucin solution was marginally small relative to other components, a simplification was used to calculate the interaction term ΔG’, as previously described by other authors [45], according to Equation (2):

G’_mix_ = G’_cp_ + ΔG’, (2)

where G’_mix_ and G’_cp_ are the elastic moduli of the mixture of the cubic phase and mucin and the cubic phase without mucin, respectively. Each rheological measurement was conducted five times according to the sequences included in rSpace software (Malvern Panalytical, Malvern, UK).

### 3.6. Turbiscan^®^ Lab Expert Stability Evaluation

A long-term stability study over time of both empty and drug-loaded precursor solutions was performed using a Turbiscan^®^ Lab Expert apparatus equipped with a Turbiscan Lab Cooler. Each precursor solution was gently placed in a cylindrical glass tube and sealed with a suitable plug. Before starting the analysis, the sample was placed into the instrument vial slot, where it was left to equilibrate at 37 ± 0.5 °C (operative temperature) for five minutes. The measurements were conducted for 3 h, during which time the integrated TurbiSoft software (Formulaction, L’Union, France) recorded alterations in delta-backscattering (ΔBS) and delta-transmission (ΔT) profiles as a function of time and vial height [48], ultimately determining the destabilization kinetic profile by measuring the Turbiscan stability index (TSI). TSI values were directly derived from a sophisticated formula by the software according to Equation (3), as previously reported [49]:

(3) $$\mathrm{TSI}=\underset{i}{\sum }\frac{\sum_{h}\left|{\mathrm{scan}}_{i}\left(h\right)-{\mathrm{scan}}_{i-1}\left(h\right)\right|}{H}$$

where scan_i_(h) is the average of backscattering recorded at each time (i) of measurement, scan_i−1_(h) is the average of backscattering for the i−1 time of measurement and H represents the sample height. The stability of samples was compared in terms of TSI, considering that TSI values can change from 0 for highly stable systems to 100 for highly unstable compounds [50,51].

### 3.7. In Vitro Drug Release Kinetic Studies

The dialysis bag method was used to evaluate the in vitro drug release from the cubic phase containing sertaconazole using a cellulose membrane with 50KDa cutoff. The drug-loaded precursor solution was injected into the dialysis bag, sealed at the extremities with suitable clamps and immersed into 100 mL of acceptor medium (SVF: methanol 75:25 *v*/*v*, pH 4.2) maintained at 37 ± 0.5 °C under continuous stirring [52]. The precursor solution rapidly transformed into a cubic phase when in contact with the aqueous medium, and at predetermined times (0, 0.5, 1, 2, 4, 6, 8, 24, 48 and 72 h), 1 mL of release medium was withdrawn, whereas the remaining medium was completely replaced with fresh acceptor medium to ensure sink conditions. The withdrawn aliquots were analyzed with an HPLC apparatus according to a previously validated method proposed by Muzaffar et al. with some modifications [52,53]. The HPLC system was equipped with a PU-4180 RHPLC quaternary pump, a UV-4070 UV-Vis Detector set to 260 nm wavelength to detect sertaconazole nitrate, an AS-4050 autosampler and an LC-NetII/ADC interface box (Jasco Europe, Lecco, Italy). The samples were eluted under isocratic conditions using an RP-C18 column (Mediterranea SEA18 column, size 250 mm × 4.6 mm, 5 µm) operating at room temperature (25 °C) under a flow of 1 mLmin^−1^, and the drug was detected at 260 nm with a retention time of around 15 min. The mobile phase was composed of a mixture of acetonitrile−0.01 M sodium phosphate buffer (pH 4) 40:60 (*v*/*v*) and was filtered through 0.22 µm polycarbonate filters prior to use; then, 20 µL of each sample was injected for analysis. Data were acquired and processed with ChromNAV v.2.04.01 software (Jasco Inc., Tokyo, Japan), using the linear equation y = 8338x + 1394 with a correlation coefficient (R^2^) of 0.9998 obtained from the analysis of standard solutions (concentration range of 0.1–20 µg/mL, *n* = 6). The plot of the calibration curve is reported as Figure S2 (Supplementary Materials).

### 3.8. In Vitro Degradation Studies

The spontaneous in vitro degradation of cubic phases was evaluated through an in vitro gravimetric method [34], using SVF as degradation medium and recording the percentage of cubic phase weight loss at predetermined times (0, 0.5, 1, 2, 4, 6, 8, 24, 48 and 72 h). Briefly, a weighted amount (*W_t0_*) of formed cubic phase was covered with 5 mL of SVF solution and thermostated at 37 ± 0.5 °C. At each time point, the beaker containing the sample was emptied, and the weight of the dry cubic phase (*W_tx_*) was recorded before refilling with fresh medium. The progressive weight loss was calculated as a percentage according to Equation (4).

(4) $$\mathrm{Weight}\;\mathrm{Loss}\;\left(\%\right)=100-\left[\left(\frac{W_{tx}}{W_{t0}}\right)\times 100\right]$$

### 3.9. Statistical Analysis

Statistical analysis between the two groups was performed by one-way ANOVA, with a *p*-value of <0.05 (*) and of <0.001 (**) considered significant. An a posteriori Bonferroni *t*-test was carried out to verify the ANOVA results.

## 4. Conclusions

In the present study, lyotropic liquid crystalline systems were designed, starting from the amphiphilic lipid GMO, capable of self-assembling into a lamellar or cubic phase. The transition of the precursor solution to a stable cubic phase was triggered only by the presence of an aqueous solution, as represented by a simulated vaginal fluid. Sertaconazole nitrate was used as a model drug within this system, as it is representative of a large class of drugs used in the treatment of vaginal infections of fungal origin that affect many women. The presence of the lipophilic drug did not, in any way, alter the ability of the precursor solution to form a stable LLC cubic phase. The anisotropic structure and the consequent formation of the isotropic cubic phase were confirmed by optical microscopic analysis. The rheological behaviors of the formulations highlighted the possibility of locally administering the precursor, owing to its low viscosity. Furthermore, the cubic phase showed a solid, three-dimensional structure, even following temperature changes. In vitro degradation studies conducted in simulated vaginal fluid at physiological pH demonstrated the resistance of the obtained LLC cubic phase, which, combined with the slow and controlled release of the drug, support its potential application for vaginal drug delivery. These findings were strongly supported by demonstrating that the formulation also interacts with mucin. The interaction between the LLC cubic phase and the mucin present in the vaginal environment could ensure a strengthening of the formulation, improving its permanence in situ and thus enhancing the functionality of the system for the proposed application.

## Figures and Tables

**Table 1:** Elastic moduli of drug-loaded cubic phase alone (G’_cp_), cubic phase/PSM mixture (G’_mix_) and calculated interaction terms (ΔG’) obtained from oscillatory frequency sweep analysis. The results are reported as mean values ± standard deviation.

Sample	Frequency (Hz)	G’_cp_ (kPa)	G’_mix_ (kPa)	ΔG’ (kPa)
Drug-loaded cubic phase + PSM 4%(*w/w*)	0.1	211 ± 18	406 ± 17	194 ± 2
	1	373 ± 6	402 ± 8	29 ± 1
	10	396 ± 4	410 ± 6	15 ± 2

**Table 2:** Representative equations, plots, slopes, intercepts and correlation coefficient (R^2^) values calculated from four kinetic models.

Kinetic Model Name	Representative Equation	Plot	Slope	Intercept	R^2^
Zero Order	$C_{t}=C_{0}+K_{0}t$	Cumulative % drug release vs. time	0.1897	2.4178	0.8836
First Order	$\log C=\log C_{0}-\frac{K_{1}t}{2.303}$	Log of cumulative % drug remaining vs. time	−0.0009	1.9894	0.8962
Higuchi	$Q=K_{H\;}\times t_{1/2}$	Cumulative % drug release vs. square root of time	1.7521	0.1747	0.9859
Korsmeyer–Peppas	$\log\left(Mt/M\infty \right)=\log K_{kp}+n\log t$	Log of cumulative % drug release vs. log of time	0.5646	0.1754	0.9587

Legend: *C_t_* is the amount of drug released at time *t*; *C*_0_ is the initial concentration of the drug at time *t = 0*; *K*_0_ is the zero-order rate constant; *C* is the percentage of drug remaining at time t; *K*_1_ is the first-order rate constant; *Q* is the cumulative amount of drug released in time *t*; *K_H_* is the Higuchi dissolution constant; *Mt* and *M∞* are the amount of drug released at time *t* and after time ∞, respectively; *K_kp_* is the Korsmeyer–Peppas release rate constant; and n is the drug release exponent.

## Data Availability

Not applicable.

## Supplementary Materials

The following are available online at www.mdpi.com/article/10.3390/molecules27196295/s1, Figure S1: Viscosity profiles of empty and drug-loaded cubic phases at 38 °C, Figure S2: Calibration curve of sertaconazole nitrate, Table S1: Mean values ± standard deviations related to TSI values recorded by Turbiscan^®^ Lab Expert during long-term stability study of precursor solutions.
