# Supplementary material for: In Situ Swelling Formulation of Glycerol-Monooleate-Derived Lyotropic Liquid Crystals Proposed for Local Vaginal Application
Source: Molecules. 2022 Sep 23;27(19):6295. doi: 10.3390/molecules27196295 (PMC9571072; doi:10.3390/molecules27196295)
Supplement: Supplementary file 1 [file molecules-27-06295-s001.zip › molecules-1917254-supplementary.pdf]

molecules

Supplementary Materials

In Situ Swelling Formulation of Glycerol Monooleate-Derived Lyotropic Liquid Crystals Proposed for Local Vaginal Application

Martine Tarsitano [1,†], Antonia Mancuso [2,†], Maria Chiara Cristiano [2], Donatella Paolino [2,*] and Massimo Fresta [1]

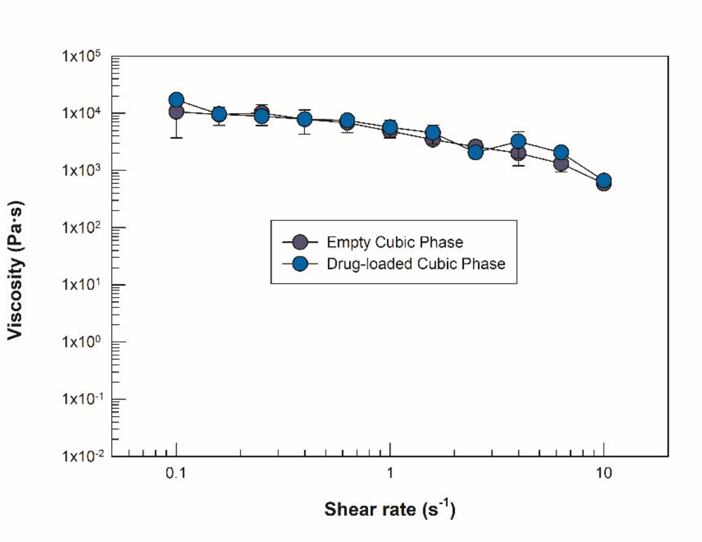

Figure S1. Viscosity profiles of empty and drug-loaded cubic phases at 38°C. The analysis were carried out at 38 ± 0.5 °C. The data are reported as mean values ± standard deviation; the error bars, if not shown, are smaller than symbol size.

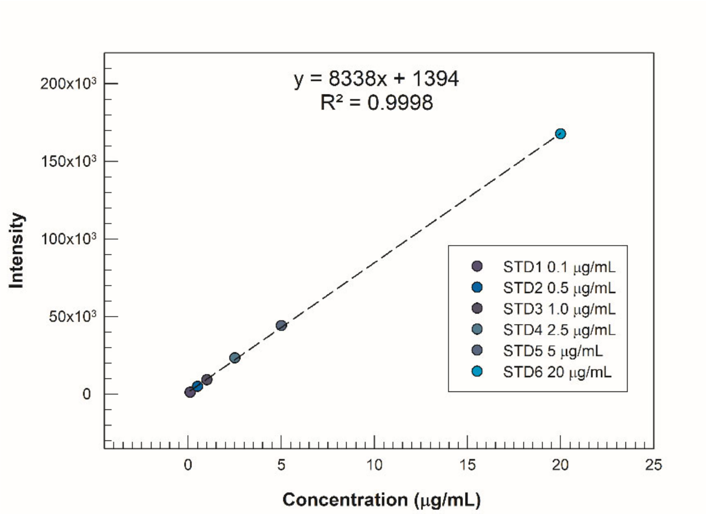

Figure S2. Calibration curve of Sertaconazole Nitrate. The calibration resulted from the analysis of different standards (STD) of the drug (n=6) in a concentration range of 0.1-20 µg/mL. At the top of the plot the regression linear equation and correlation coefficient R^2 are shown.

Table S1. Mean values ± standard deviations related to TSI values recorded by Turbiscan® Lab Expert during long-term stability study of precursor solutions.

Time	Empty Precursor Solution	Drug Loaded Precursor Solution
0 s	0.0 ± 0	0.0 ± 0
30 s	0.0 ± 0	0.0 ± 0
15 min	0.3 ± 0.05	0.4 ± 0.2
30 min	0.6 ± 0.1	0.8 ± 0.2
1 h	1.2 ± 0.2	1.5 ± 0.2
1 h 30 min	1.7 ± 0.2	2.2 ± 0.1
2 h	2.4 ± 0.1	2.8 ± 0.05
2 h 30 min	2.9 ± 0.05	3.3 ± 0.05
3 h	3.4 ± 0.05	3.9 ± 0.05
